# Hypoxia-induced Tie1 drives stemness and cisplatin resistance in non-small cell lung carcinoma cells

**DOI:** 10.1186/s12935-020-01729-3

**Published:** 2021-01-18

**Authors:** Chaojie Li, Nannan Yang, Zhijin Chen, Ning Xia, Qungang Shan, Ziyin Wang, Jian Lu, Mingyi Shang, Zhongmin Wang

**Affiliations:** 1 Department of Radiology, Ruijin Hospital Luwan Branch, Shanghai Jiao Tong University School of Medicine, No. 149 Chongqing South Road, Shanghai, 200025 China; 2 Department of Interventional Radiology, Tongren Hospital, Shanghai Jiao Tong University School of Medicine, 1111 Xianxia Road, Shanghai, 200336 China; 3 Department of Interventional Radiology, Ruijin Hospital, Shanghai Jiao Tong University School of Medicine, No. 197 Ruijin Er Road, Shanghai, 200000 China

**Keywords:** Tie1, Hypoxia, Stemness, HIF-1α, Cisplatin resistance

## Abstract

**Background:**

Drug resistance and metastasis involving hypoxic tumor environments and persistent stem cell populations are detrimental to the survival of patients with non-small cell lung carcinoma (NSCLC). Tie1 is upregulated in hypoxia and is believed to counteract the effectiveness of platinum agents by promoting the stemness properties in cells. We have investigated the association of Tie1 with HIF-1α and cisplatin resistance in NSCLC cell lines.

**Methods:**

The expression of Tie1 in a pulmonary microvascular endothelial cell line (HPMEC) and NSCLC cell lines was detected using qRT-PCR and western blotting. The effect of Tie1 on cell stemness and migration was examined by sphere-forming and transwell assays in NSCLC cells with Tie1 silenced. The regulation of Tie1 by HIF-1α was evaluated by a dual-luciferase reporter assay and chromatin immunoprecipitation.

**Results:**

We found that hypoxia could induce stemness and cisplatin resistance in vitro. Tie1 was expressed at low levels in NSCLC cells when compared with human pulmonary microvascular endothelial cells, however, its expression was increased by hypoxia. Additionally, Tie1 knockdown could reduce stemness properties and increase sensitivity to cisplatin in vitro and in a xenograft mouse model. The promoter of Tie1 contains two predicted hypoxia-response elements (HREs). We mutated both HRE sites and conducted chromatin immune-precipitation and promoter luciferase reporter assays and were able to conclude that the induction of Tie1 by hypoxia was HIF-1α-dependent.

**Conclusions:**

Our findings indicated that Tie1 is upregulated in a hypoxic environment by HIF-1α and contributes to tumorigenesis and cisplatin resistance through the promotion of stemness in NSCLC cells.

## Background

Although the incidence of lung cancer has decreased considerably over the last decade, it is still responsible for greater mortality than breast, colorectal, and prostate cancers combined and remains the leading cause of cancer-related deaths globally [1, 2]. The World Health Organization has classified lung cancer as either small cell or non-small cell depending on histology [3]. However, the majority of lung cancers (85%) are non-small cell lung carcinoma (NSCLC) and include adenocarcinoma, squamous cell carcinoma, and large cell carcinoma [4]. The low survival rate in lung cancer has been attributed to an advanced stage and metastases at diagnosis [5]. In addition, drug resistance can also contribute to high mortality [6], with cancer stem cells (CSCs) emerging as a possible instigator in tumor progression, metastasis, and drug resistance [7]. The drugs used most often in first-line chemotherapy for NSCLC are platinum agents such as cisplatin or carboplatin with paclitaxel, gemcitabine, docetaxel, vinorelbine, irinotecan, or pemetrexed [8]. However, acquired resistance or intolerance to these drugs has led to a need for therapies that are more targeted [9].

Drug resistance is attributed to high mortality in several other cancers besides NSCLC, including ovarian, breast, and colorectal cancer [10]. In ovarian cancer, Tie1, which forms part of the tyrosine kinase with immunoglobulin and epidermal growth factor homology domains (TIE) signaling pathway, was found to be involved in platinum-resistance and its high expression was correlated with a significantly poor prognosis [11]. Ishibashi et al.[11] found that the overexpression of Tie1 upregulates nucleotide excision repair to counteract the DNA-adduct damage and subsequent apoptosis caused by cisplatin and other platinum agents. Tie1 encodes a tyrosine kinase receptor that not only has involvement in drug resistance but may also function in tumorigenesis [12]. In colorectal cancer, Tie1-positive cells were found in populations with CSC characteristics and were able to express the stem cell marker LGR5 [12]. In NSCLC, a female group of patients with adenocarcinoma and high expression of Tie1 had lower overall survival than those with a low expression of Tie1 [13]. In a recent phase II trial with taxane–bevacizumab combination chemotherapy, elevated levels of ANG2 and Tie1 in the blood plasma of patients with metastatic breast cancer were associated with shorter overall survival than baseline levels [14]. During hypoxia and inflammation, Tie1 was found to induce the antagonistic disruptive form of ANG2 [15, 16].

Hypoxic conditions are thought to promote drug resistance because the maintenance of CSCs is dependent on low oxygen and hypoxia-inducible factors (HIF) 1α and 2α [17]. HIF-1α promotes tumorigenesis during hypoxia through the coordination of various transcription factors and subsequent activation of signaling pathways by interacting with hypoxia-response elements (HREs) in promoters [18–20]. Several diseases are believed to result from the dysregulation of hypoxia-related pathways by HIF-1α through HRE sites [21, 22]. For instance, Twist and BMI1 are thought to influence epithelial cell–mesenchymal transition during renal fibrogenesis when upregulated in hypoxic conditions by HIF-1α through HRE sites [21, 23].

In this study, we investigate the stemness properties of NSCLC cells in response to hypoxia by the expression of hypoxic and stem cell-related markers and whether this influences resistance to cisplatin. In addition, we have identified two sites with consensus HRE binding sequence in the promoter of Tie1. After mutating the HRE sites, we have assessed the involvement of HIF-1α on the induction of Tie1 in NSCLC cells and the consequences on drug resistance and the stemness of cells.

## Materials and methods

### Cell culture and hypoxic culture conditions

The NSCLC cell line NCI-H520 was purchased from the American Type Culture Collection (Manassas, VA, USA). The A549, NCI-H460, and NCI-H1975 cell lines were purchased from the Culture Collection of the Chinese Academy of Sciences (Shanghai, China). The NSCLC cell lines were maintained in RPMI 1640 (Thermo Fisher Scientific, Waltham, MA, USA) supplemented with 5% fetal bovine serum (FBS) and 5 mg/mL penicillin/streptomycin at 37 °C. Human pulmonary microvascular endothelial cells (HPMEC) were purchased from ScienCell Research Laboratories (San Diego, CA, USA) and maintained in endothelial cell medium (ScienCell) supplemented with 10% FBS and 5 mg/mL penicillin/streptomycin at 37 °C. Cells under normoxic conditions were incubated at 21% O_2_ and 5% CO_2_. Cells under hypoxic conditions were incubated at 1% O_2_ and 5% CO_2_.

### Western blotting analysis

To extract proteins for western blot analysis, cells were first lysed in RIPA buffer supplemented with proteinase inhibitors (Pierce Biotechnology, Rockford, IL, USA). Equivalent concentrations of protein measured by a BCA Protein Assay Kit (Thermo Fisher Scientific) were separated by SDS-PAGE and transferred to PVDF membranes (Millipore, Burlington, MA, USA). Membranes were blocked with 5% non-fat milk for 1 h and then incubated overnight at 4 °C with primary antibodies against Tie1 (1:1000, Abcam, Cambridge, UK), BMI-1 (1:1000, Abcam), LGR5 (1:1000; Bioss, Beijing, China), CD44 (1:1000, Cell Signaling Technology, Danvers, MA, USA), HIF-1α (1:2000, Abcam), β-actin (1:5000, Abcam), and HA-tag (1:3000, Proteintech, Wuhan, China). Following incubation for 1 h with HRP-conjugated secondary antibodies (Cell Signaling Technology), immunoreactive bands were visualized with an enhanced chemiluminescence detection system (Thermo Fisher Scientific). The relative intensities of target proteins were quantified by ImageJ software (National Institutes of Health, Bethesda, MD, USA).

### Quantitative reverse transcription PCR (qRT-PCR)

Total RNA was extracted from cells with Trizol reagent (Invitrogen, Carlsbad, CA, USA). A PrimeScript RT reagent kit (Takara, Kyoto, Japan) was used to synthesize cDNA and an SYBR Green Realtime PCR Premix (Takara) was used with the primer sequences: Tie1, Forward 5′-TTGTGCCCCTGGTCATTTTG-3′, Reverse 5′-TCCAGTTCTGAGGCCATGTT-′; GAPDH, Forward 5′-TCAAGAAGGTGGTGAAGCAGG-3′, Reverse 5′-TCAAAGGTGGAGGAGTGGGT-3′. The relative abundance of mRNA was normalized to GAPDH and the 2^−ΔΔCT^ method was used to analyze expression levels.

### Cell viability assays

Cells were incubated for 48 h with a gradient of cisplatin concentrations (0–32 μM; Sigma-Aldrich, St. Louis, MO, USA). Cell viability was assessed using a Cell Counting Kit-8 (CCK-8, Dojindo Laboratories, Kumamoto, Japan) according to the manufacturer’s instructions. Briefly, cells (~ 3 × 10^3^) were grown under normoxic or hypoxic conditions in 96-well plates with or without cisplatin for up to 48 h. CCK-8 solution (10 μL) was added to each well and after 2 h incubation at 37 °C, formazan dye was measured at 450 nm absorbance on a microplate reader (Bio-Rad Laboratories, Hercules, CA, USA) and compared to a standard curve.

### Sphere formation assay

To assess the formation of spheres, cells (1 × 10^6^ per well) preincubated under normoxic or hypoxic conditions were seeded in six-well plates in complete medium. Sphere formation was induced by the addition of 2% B27, 20 ng/mL basic fibroblast growth factor, and 20 ng/mL epidermal growth factor in ultralow attachment six-well plates. After 7 days of incubation, spheres with a diameter greater than 50 μM were counted at ×40 magnification.

### Cell migration assay

Cell migration was assessed with the aid of 24-well plate 8-mm pores Transwell inserts (Millipore). Cells (1 × 10^4^) were plated into the top chamber and allowed to migrate into the lower chamber for 24 h. Migrated cells were stained with 0.2% crystal violet and counted at ×40 magnification.

### Immunofluorescence (IF) staining

Cells were fixed with 4% paraformaldehyde, washed with PBS, and then blocked with10% goat serum. They were then incubated with HIF-1α (1:200, Abcam) antibodies overnight at 4 °C. After incubation, cells were washed twice with PBS and stained with Cy3 (red)-conjugated secondary antibody for a further 2 h at 37 °C. Surplus antibody was removed by washing before obtaining images with an Olympus microscope (Olympus, Tokyo, Japan) at × 40 magnification. Images were captured with a DP50 camera and DP50 software (Olympus).

### Plasmid constructs, lentivirus production, and transfection

The stable knockdown of Tie1 was obtained by transfecting A549 and NCI-H1975 cells with lentiviral constructs containing short hairpin RNA (shRNA) sequences. The target sequences 5′-GAGAACCTAGCCTCCAAGATT-3′ of Tie1 shRNA were cloned into the vector GV248 (Genechem, Shanghai, China). A non-silencing scrambled shRNA was used as a negative control (5′-GGCAAGACATACGCTCTCATA-3′). Small interfering RNA (siRNA) against human HIF-1α (target sequences: 5′-GACGATCATGCAGCTACTACA-3′) and negative control (5′-GGCACTATCCAACGGTAATCA-3′) were synthesized by GenePharma (Shanghai, China) and transfected into A549 and NCI-H1975 cells. To construct a HIF-1α overexpression vector, the full-length human HIF-1α was cloned into a pcDNA3-HA vector. The dominant-negative HIF-1α construct lacking DNA binding and activation domains was generated by PCR and cloned into a pcDNA3-HA vector and transfected into A549 and NCI-H1975 cells. All constructs were confirmed by sequencing and all cells were transfected with vectors or siRNA using Lipofectamine 2000 Reagent (Invitrogen) following the manufacturer’s instructions.

### Promoter luciferase reporter assays

The HRE sites in the promoter of Tie1 were assessed using the Dual-Luciferase Reporter Assay System (Promega, Madison, WI, USA). The consensus sequence of the HRE sites in the 1500-bp fragment of the human Tie1 promoter (from − 1500 to − 1 bp relative to the translation start site) were mutated by substituting CG with AT. The DNA fragment was then cloned into vector pGL3 to generate the construct pGL3-Tie1-Luc. The luciferase reporter vector containing the Tie1 promoter was then transfected into A549 cells together with the pRL-TK Renilla luciferase plasmids. Cells were transfected using Lipofectamine 2000 Reagent (Invitrogen) following the manufacturer’s instructions. Luciferase activity was measured after 48 h incubation under hypoxic conditions.

### Chromatin immunoprecipitation (ChIP) assay

Binding between the promoter region of Tie1 and HIF-1α was assessed used a ChIP assay kit (Millipore) following the manufacturer’s instructions. Briefly, A549 cells were incubated under hypoxic conditions for 24 h and then sonicated to obtain fragmented DNA. Chromatin was immunoprecipitated with anti-HIF-1α (Novus Biologicals, Littleton, CO, USA) or rabbit IgG (Sigma-Aldrich). The region in the Tie1 promoter containing the HIF-1α-binding site (5′-CTCGTG-3′, from − 1032 to − 1038 bp relative to the translation start site) was detected and amplified by PCR using the following primers: forward 5′-CATCCCAACCATTCCATTCCG-3′ and reverse 5′-TTCCCAGAACGGAACAAGACC-3′.

### Mouse xenograft model

All animal experiments were performed in accordance with the guidelines of the Laboratory Animal Ethical Committee at Shanghai Jiao Tong University (Approval number B-2018–010). BALB/c male nude mice (4–6 weeks old) were obtained from Shanghai SLAC Laboratory Animal Co., Ltd (Shanghai, China) and acclimatized for 1 week in specific pathogen-free conditions with sterile food and water. A549 cells stably transfected by scramble or Tie1-shRNA lentiviruses were subcutaneously injected into the right flank of the nude mice. Mice were randomly divided into control (saline) or cisplatin groups (n = 6, 2 mg/kg body weight delivered by intraperitoneal injection three times a week over 3 weeks). Tumor growth was measured every 5 days. The tumor volume was determined by the formula: Volume (mm^3^) = Length × (Width)^2^. At the end of the experiment mice were euthanized by cervical decapitation and tumors were excised and weighed. Hematoxylin and eosin (H&E) staining and immunohistochemistry analysis (IHC) of paraffin sections from xenograft samples was performed using antibodies against Ki67 (1:500, Cell Signaling Technology). For the limiting dilution tumorigenicity assay, each nude mouse was injected with different concentrations of cells (1 × 10^6^, 1 × 10^5^, 1 × 10^4^, or 1 × 10^3^ cells in 100 μl DMEM, n = 5) subcutaneously in the flank. The volume of the tumors was observed and recorded after 5 weeks.

### Statistical analysis

All data were analyzed using the statistical software package GraphPad Prism 7.0 (GraphPad, San Diego, CA, USA). Statistical significance between two groups was determined using the Student’s *t* test, and comparisons among more than two groups were performed using analysis of variance (ANOVA). All in vitro experiments were repeated at least three times independently. Data are presented as the mean ± standard deviation (SD) unless otherwise stated. P < 0.05 was considered significant.

## Results

### Increased Tie1 expression in hypoxic lung cancer cells contributes to a reduction in cisplatin sensitivity

**Fig. 1:**
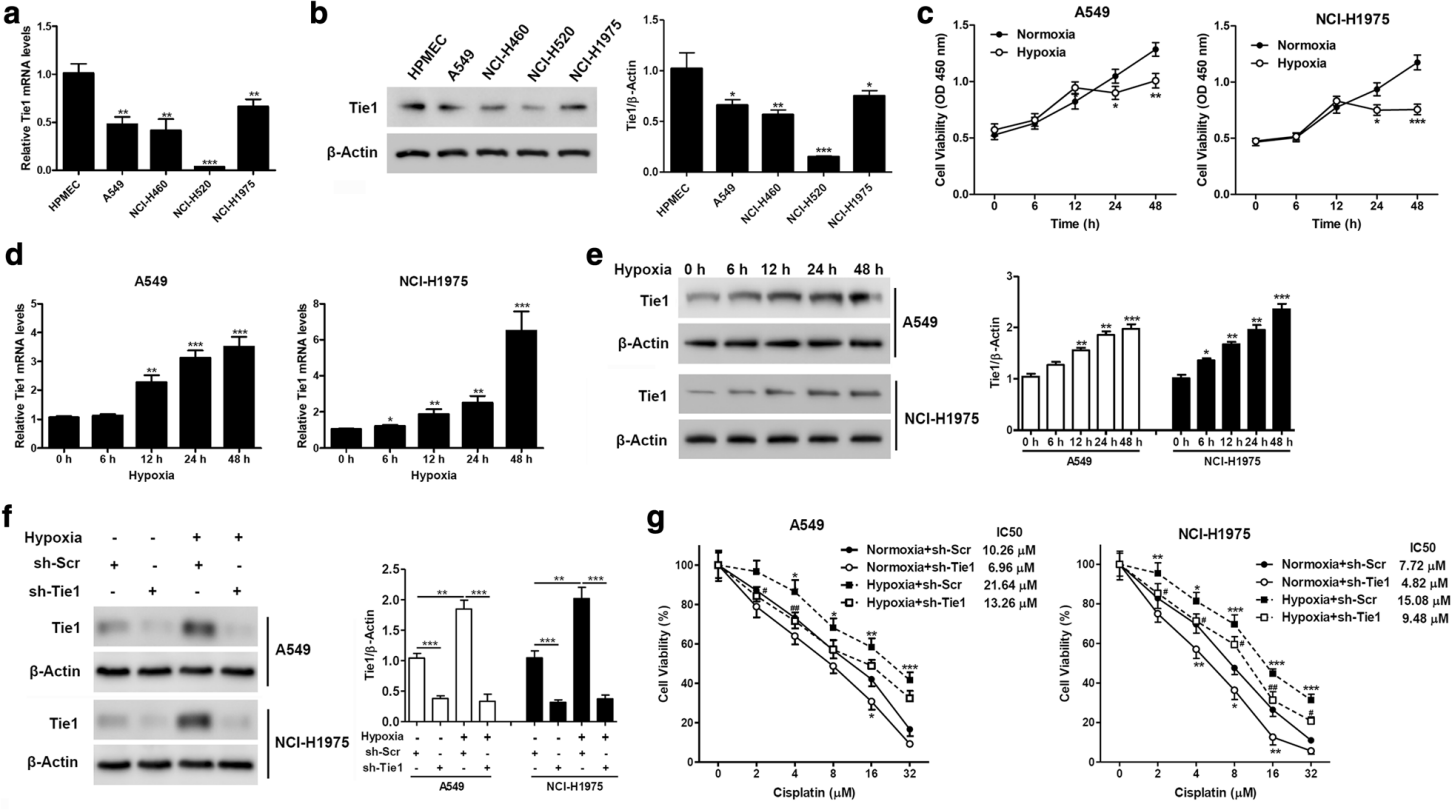
Hypoxia-enhanced Tie1 expression in human lung cancer cells contributes to reduced cisplatin sensitivity. **a** and **b**, Tie1 mRNA levels (**a**) and protein levels (**b**) in human pulmonary microvascular endothelial cells (HPMECs) and human NSCLC cell lines were detected by qRT-PCR and western blot, respectively. *P < 0.05, **P < 0.01, ***P < 0.001. Relative protein levels quantified by ImageJ and normalized to β-actin. (n = 3). **c** Cell viability of A549 cells, and NCI-H1975 cells exposed to hypoxia for 0, 6, 12, 24, and 48 h. **d** and **e**, A549 cells, and NCI-H1975 cells were exposed to hypoxia for 0, 6, 12, 24, and 48 h. mRNA levels (**d**) and protein levels (**e**) were detected by qRT-PCR and western blotting, respectively. Relative protein levels quantified by ImageJ and normalized to β-actin. *P < 0.05, **P < 0.01, ***P < 0.001. (n = 3). **f** A549 cells and NCI-H1975 cells stably transfected by scramble or Tie1-shRNA lentiviruses were incubated under hypoxic conditions for 48 h. Proteins were analyzed using western blotting. Relative protein levels quantified by ImageJ and normalized to β-actin. **P < 0.01, ***P < 0.001. (n = 3). **g** A549 cells and NCI-H1975 cells stably transfected by scramble or Tie1-shRNA lentiviruses were treated with cisplatin under normoxic or hypoxic conditions for 48 h. Cell viability was assessed by CCK-8 assays. *P < 0.05, **P < 0.01, ***P < 0.001 vs Normoxia + sh-Scr, ^#^P < 0.05, ^##^P < 0.01 vs Hypoxia + sh-Scr, using two-way ANOVA followed by Bonferroni post hoc test. (n = 3)

To assess the activity of Tie1, we first compared its expression in a pulmonary microvascular endothelial cell line (HPMEC) and NSCLC cell lines (A549, NCI-H460, NCI-H520, and NCI-H1975). Tie1 mRNA and protein levels were significantly higher in HPMECs than in NSCLC cells (Fig. 1a, b). Expression in the A549, NCI-H460, and NCI-H1975 cells was at a similar level, with NCI-H1975 slightly higher than the other two cell lines, whereas the expression in NCI-H520 cells was almost undetectable. We selected cell lines A549 and NCI-H1975 to investigate whether hypoxia may influence the expression of Tie1. At 24 h, cell viability was significantly lower in both cell lines under hypoxic conditions compared to normoxic conditions, with viability remaining lower for 48 h during hypoxia (Fig. 1c). After 12 h under hypoxic conditions, the expression of Tie1 significantly increased in both cell lines and continued to increase until a highly significant level was reached (Fig. 1d, e). These results indicate that under normoxic conditions, Tie1 expression is lower in NSCLC cells than in normal dividing cells but under hypoxic conditions, such as that found in tumor microenvironments, the expression of Tie1 significantly increases. To analyze whether the high expression of Tie1 could influence drug resistance, A549 and NCI-H1975 cells transfected with scramble or Tie1-shRNA lentiviruses were treated with cisplatin under normoxic or hypoxic conditions for 48 h (Fig. 1f, g). Initially, cells cultured under hypoxia were resistant to cisplatin. However, it was clear that the cells with Tie1 silenced had a significantly decreased cell viability and were more sensitive to cisplatin, under hypoxic conditions. Overall, these results indicate that hypoxia increases drug resistance in NSCLC cells and that Tie1 expression promotes a more resistant phenotype.

### Tie1 knockdown impairs hypoxia-induced cancer stemness properties

**Fig. 2:**
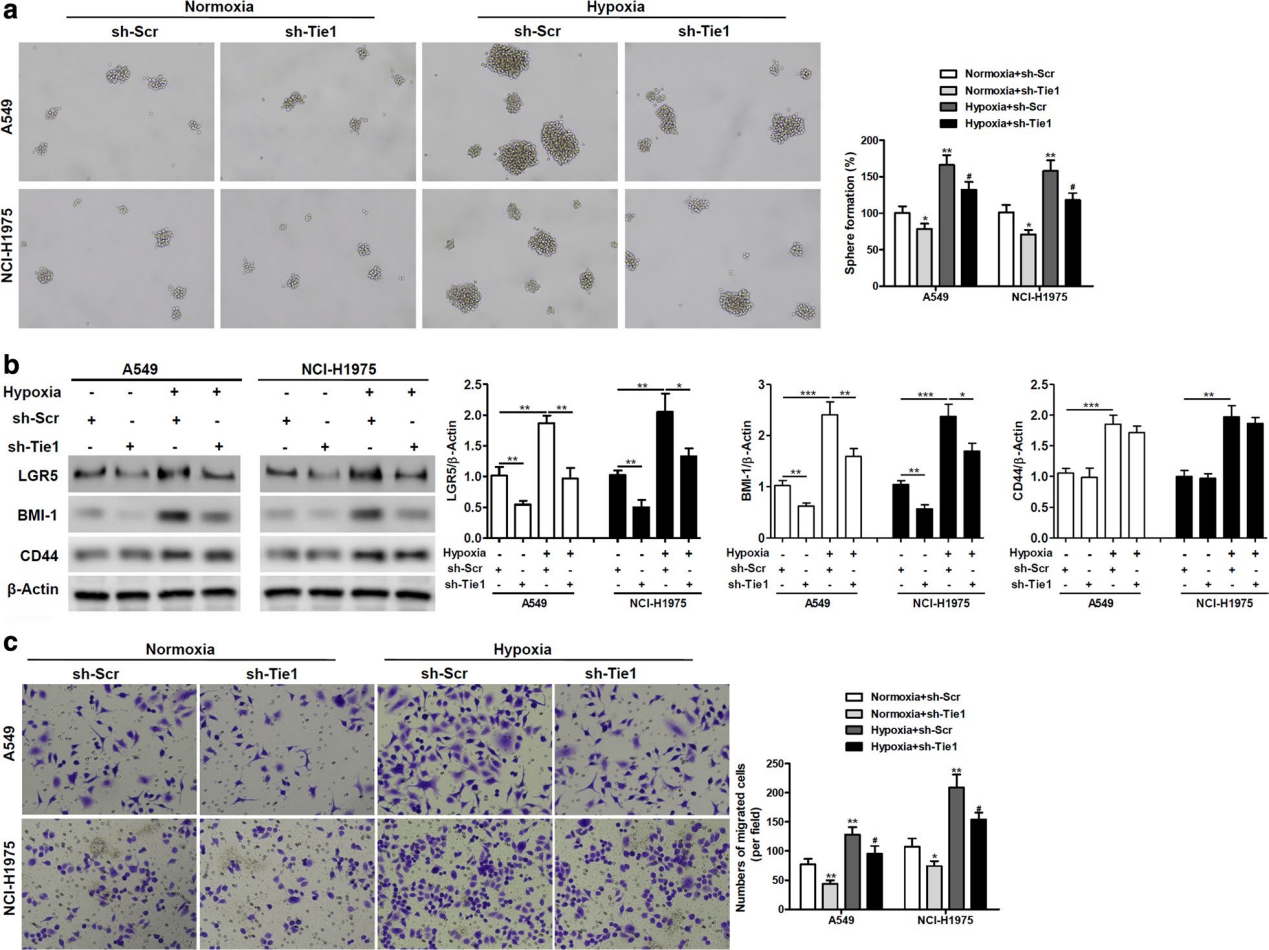
Tie1 knockdown constrains hypoxia-induced cancer stemness properties. **a** Representative image of spheres after treatment. A549 cells and NCI-H1975 cells transfected with scramble or Tie1-shRNA lentiviruses were exposed to normoxic or hypoxic conditions for 48 h. The ability of the cells to form spheres was investigated by a sphere-forming assay. The percentage of sphere formation (diameter > 50 μm) is shown in the bar graph. *P < 0.05, **P < 0.01 vs Normoxia + sh-Scr, ^#^P < 0.05 vs Hypoxia + sh-Scr. (n = 3). **b** The expression levels of stemness-related genes were determined by western blot analysis. Relative protein levels quantified by ImageJ and normalized to β-actin. *P < 0.05, **P < 0.01, ***P < 0.001. (n = 3). **c** Migration ability of A549 and NCI-H1975 cells transfected by scramble or Tie1-shRNA lentiviruses incubated under normoxic or hypoxic conditions for 48 h were evaluated by Transwell assays. The numbers of migrated cells are shown in the bar graph. *P < 0.05, **P < 0.01 vs Normoxia + sh-Scr, ^#^P < 0.05 vs Hypoxia + sh-Scr. (n = 3)

Having established that Tie1 knockdown reduces cell viability under hypoxia we investigated whether hypoxia-induced cell stemness properties are impaired. A sphere-forming assay indicated that cell stemness was reduced in A549 and NCI-H1975 cells with Tie1 knockdown after incubation for 48 h in hypoxic conditions (Fig. 2a). Hypoxic conditions induced a higher level of sphere formation than normoxic conditions. We also determined the expression levels of stemness-related markers by western blot analysis. The expression levels of LGR5, BMI-1, and CD44 were increased with hypoxia but LGR5 and BMI-1 levels reduced significantly and CD44 expression decreased slightly when Tie1 was knocked down (Fig. 2b). This indicates that a number of pathways could be involved in the response to hypoxia and the stemness of cells. Moreover, hypoxia increases the migration of NSCLC cells and Tie1 knockdown can reduce the number of migratory cells under normoxic and hypoxic conditions (Fig. 2c). Therefore, Tie1 expression can influence the stemness of NSCLC cells, especially under hypoxic conditions.

### Tie1 knockdown enhances the anti-tumor effect of cisplatin in vivo

**Fig. 3:**
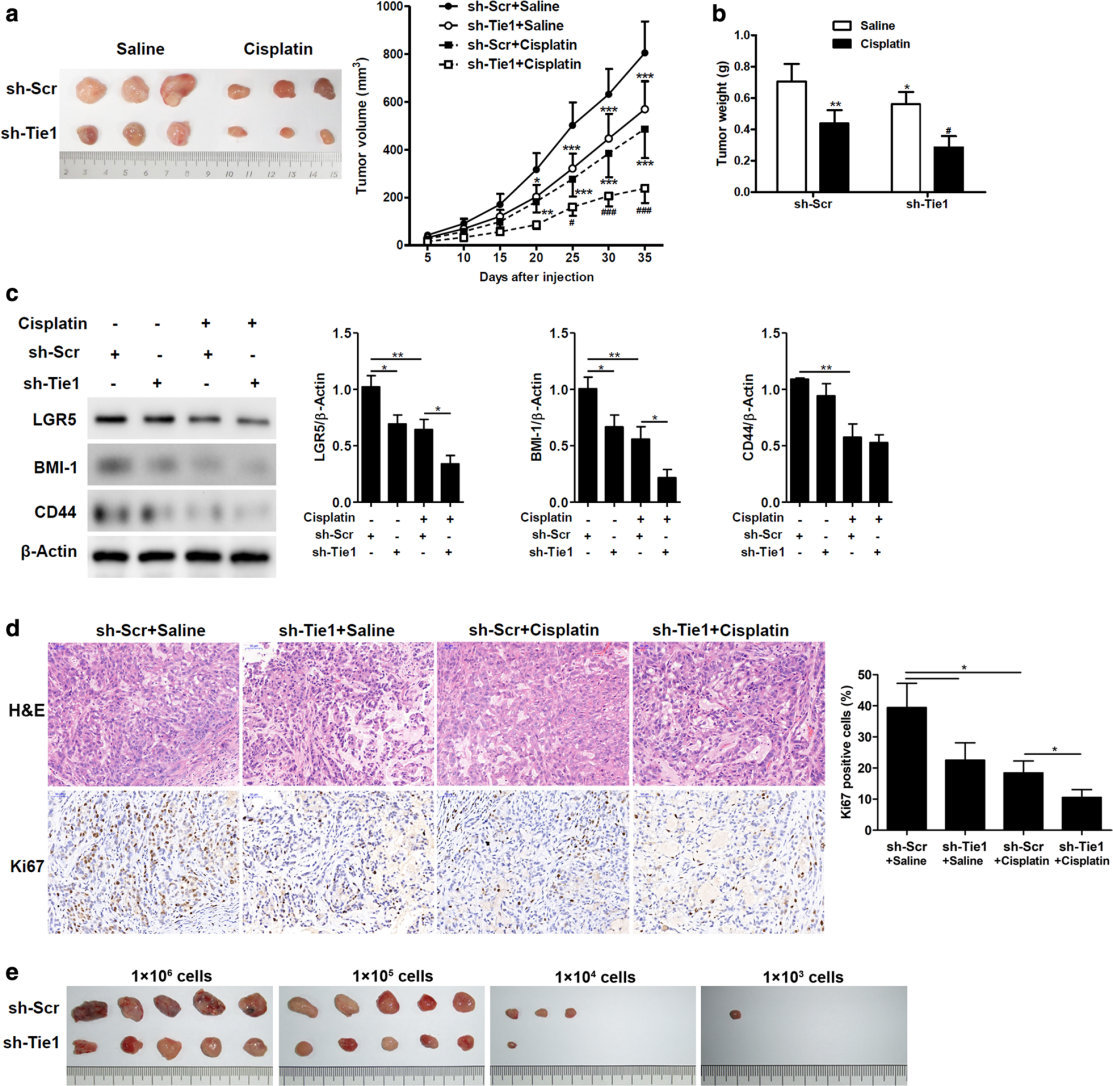
Tie1 knockdown enhances the anti-tumor effect of cisplatin in vivo. **a** A549 cells transfected by scramble or Tie1-shRNA lentiviruses were subcutaneously injected into the right flank of nude mice. Representative images of xenografts at 35 days post-treatment and tumor growth curves delineated on the basis of volume measured every 5 days. *P < 0.05, **P < 0.01, ***P < 0.001 vs sh-Scr + Saline, ^#^P < 0.05, ^###^P < 0.001 vs sh-Scr + Cisplatin, using two-way ANOVA followed by Bonferroni post hoc test. (n = 6). **b** Tumor weights of xenografts were evaluated. *P < 0.05, **P < 0.01 vs sh-Scr + Saline, ^#^P < 0.05 vs sh-Scr + Cisplatin. (n = 6). **c** Expression levels of stemness-related genes were determined by western blot analysis. Relative protein levels quantified by ImageJ and normalized to β-actin. *P < 0.05, **P < 0.01. (n = 3). **d** Representative images of H&E staining and immunohistochemical staining of Ki67 expression in xenograft tumor tissues. Scale bar = 50 μm. Relative quantification of the IHC staining was performed using ImageJ software. *P < 0.05. (n = 3). **e** Images of tumor formation in the limiting dilution tumorigenicity assay. Nude mouse was injected with different concentrations of cells subcutaneously in the flank (n = 5). The volume of the tumors was recorded after 5 weeks

In a murine xenograft model, A549 cells stably transfected by scramble or Tie1-shRNA lentiviruses were subcutaneously injected into the right flank of nude mice. Mice received either cisplatin or saline control (Fig. 3a, b). Tie1 knockdown was found to enhance the anti-tumor effect of cisplatin in vivo as demonstrated by the reduced volume of tumors. The expression levels of stemness-related genes were also reduced when Tie1 was knocked down in tumors (Fig. 3c). Immunohistochemical staining of the cell division marker Ki67 in xenograft tumor tissues confirmed that cells become more sensitive to cisplatin and cell division is reduced when the expression of Tie1 is lowered (Fig. 3d). To establish the role of Tie1 in cancer stem cell, we performed limiting dilution tumorigenicity assay, further found that A549 cells transfected with sh-Tie1 formed smaller subcutaneous tumors (Fig. 3e). The results in the mouse xenograft model support the in vitro findings that Tie1 promotes the stemness of cells and reduces the sensitivity to cisplatin.

### Hypoxia-mediated induction of Tie1 is HIF-1α-dependent

**Fig. 4:**
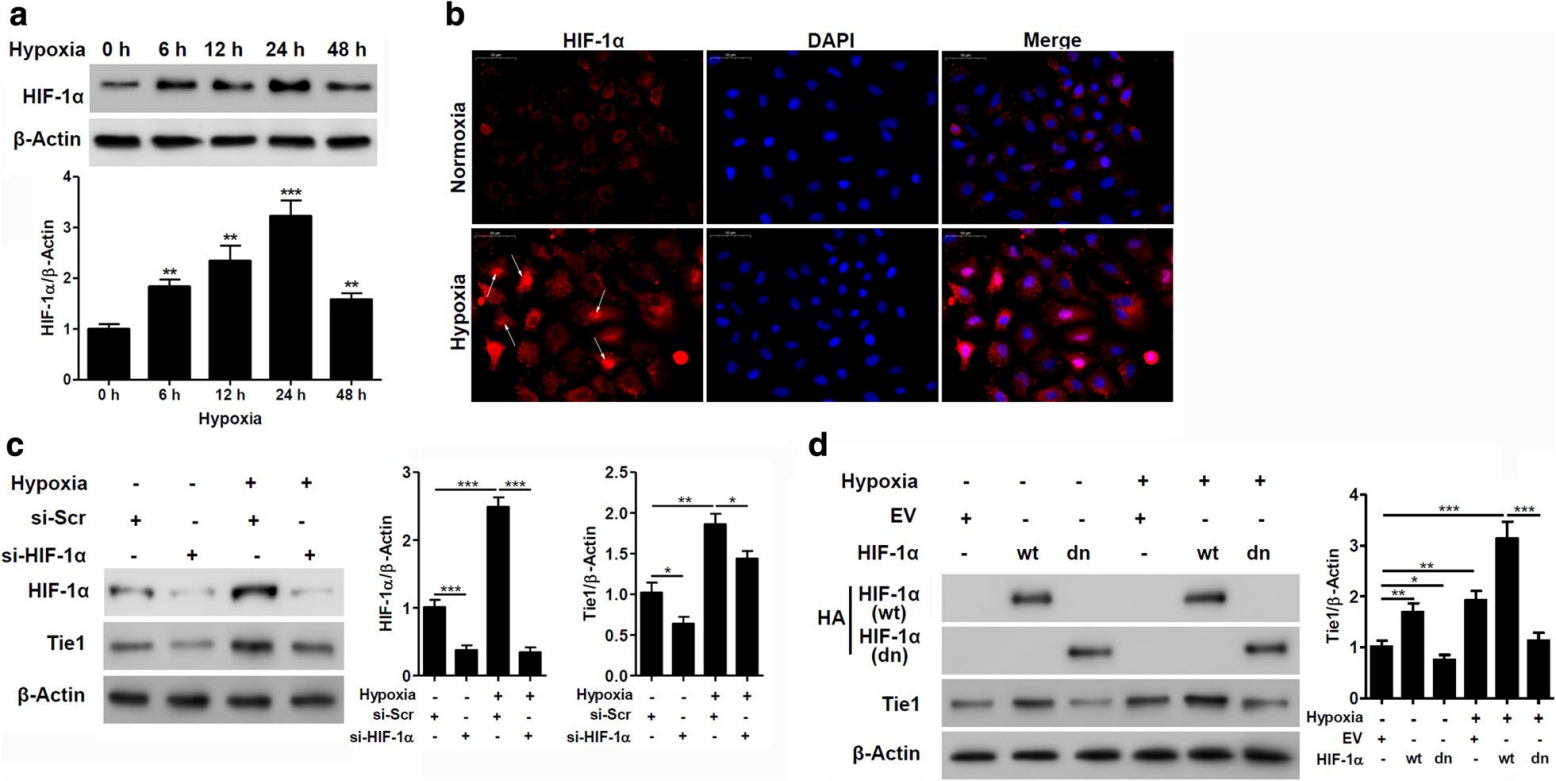
Hypoxia-mediated induction of Tie1 expression is HIF-1α-dependent. **a** A549 cells were incubated under different hypoxic conditions, and the expression levels of HIF-1α were determined by western blot analysis. Relative protein levels quantified by ImageJ and normalized to β-actin. **P < 0.01, ***P < 0.001. (n = 3). **b** Immunofluorescence staining of HIF-1α (red) and DAPI (blue) in A549 cells under normoxic or hypoxic conditions. Scale bar = 50 μm. Arrows indicate nuclear accumulation of HIF-1α. **c** Tie1 expression in A549 cells transfected with siRNA against HIF-1α or scramble (si-Scr) treated with hypoxia. Relative protein levels quantified by ImageJ and normalized to β-actin. *P < 0.05, **P < 0.01, ***P < 0.001. (n = 3). **d** Tie1 expression in A549 cells transfected with wild-type (wt) or dominant-negative (dn) HA-tagged HIF-1α cultured under hypoxic conditions. Relative protein levels quantified by ImageJ and normalized to β-actin. *P < 0.05, **P < 0.01, ***P < 0.001. (n = 3)

To characterize the involvement of hypoxia in the induction of Tie1, we measured the level of HIF-1α expression in A549 cells under hypoxic conditions. After 24 h, HIF-1α reaches a significantly higher level of expression than in normoxic conditions (Fig. 4a). The elevation of HIF-1α transcription factor levels and its translocation to the nucleus by hypoxia are demonstrated by immunofluorescence staining (Fig. 4b). To validate if the levels of Tie1 are influenced by HIF-1α, we measured the level of both proteins under hypoxia. We found that the expression of both proteins was increased by hypoxia but the level of Tie1 is also reduced when HIF-1α is downregulated (Fig. 4c). Moreover, when A549 cells were transfected with a dominant-negative version of HIF-1α, containing mutated binding and activation sites, Tie1 expression under hypoxia was reduced compared to cells transfected with wild-type (wt) HIF-1α (Fig. 4d). Taken together, these results indicate that the hypoxia-mediated induction of Tie1 expression is HIF-1α-dependent.

### HIF-1α regulates Tie1 expression through direct binding to its promoter

**Fig. 5:**
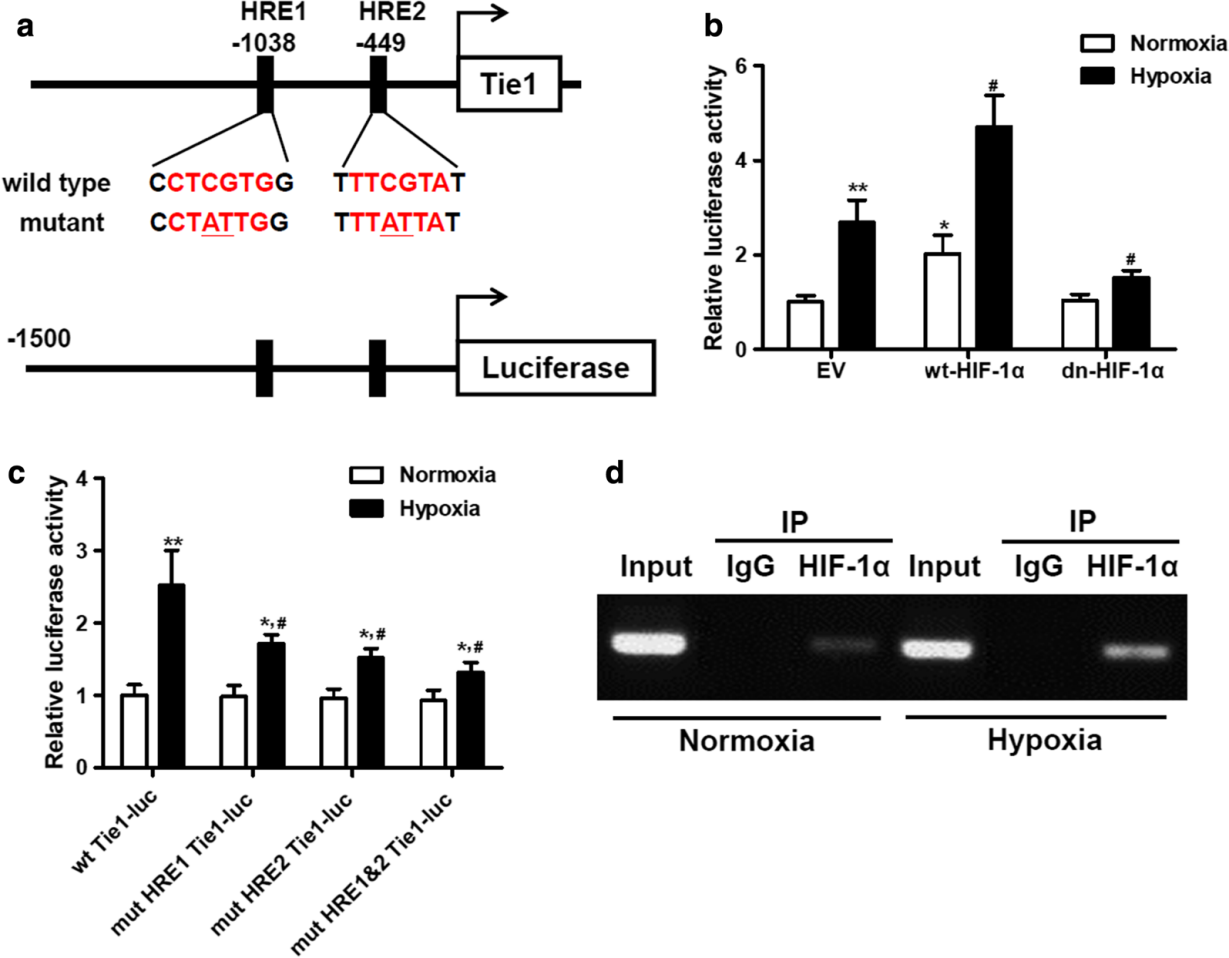
HIF-1α directly binds to the Tie1 promoter. **a** Diagram showing the two predicted HIF-1α binding sites on the Tie1 promoter and mutagenesis information of each HRE site. **b** A549 cells were co-transfected with pGL3-Tie1-Luc and wild-type (wt) or dominant-negative (dn) HIF-1α followed by hypoxic incubation, and luciferase activity was measured at 48 h after hypoxia. *P < 0.05, **P < 0.01 vs Normoxia + EV, ^#^P < 0.05 vs Hypoxia + EV. (n = 3). **c** A549 cells were transfected with pGL3-Tie1-Luc or HRE mutant constructs containing the HRE1 mutation and/or HRE2 mutation, followed by hypoxic incubation, and luciferase activity was measured at 48 h after hypoxia treatment. *P < 0.05, **P < 0.01 vs Normoxia + wt Tie1-Luc, ^#^P < 0.05 vs Hypoxia + wt Tie1-Luc. (n = 3). **d** A549 cells were incubated under hypoxic or normoxic conditions for 24 h. Cell lysate was collected for the ChIP analysis of HIF-1a binding to Tie1 promoter DNA

To understand the regulation of Tie1 further, in silico analysis was performed and we identified two sites that shared homology with the HIF-1α–binding consensus sequence BDCGTV (B = C/T/G, D = A/G/T, V = G/C/A). The promoter of Tie1 contains two predicted HIF-1α binding sites HRE1 and HRE2. We mutated both sites and conducted a luciferase activity assay to establish whether the sites could be involved in the activation by HIF-1α (Fig. 5a). Luciferase activity was significantly increased under hypoxia, wt-HIF-1α significantly increased the basal and hypoxia-induced elevation of luciferase activity, but HIF-1α-with a dominant negative mutation effectively inhibited it (Fig. 5b). In A549 cells transfected with pGL3-Tie1-Luc or HRE mutant constructs, the wt promoter induced a higher level of luciferase activity than the promoter with either HRE1 and/or HRE2 mutations under hypoxia (Fig. 5c). ChIP analysis was used to confirm the direct binding of HIF-1α to the Tie1 promoter DNA (Fig. 5d). These findings indicate that both HRE sites are required for the induction of Tie1 by HIF-1α.

### HIF-1α controls the stemness of NSCLC cell by regulating Tie1 expression

**Fig. 6:**
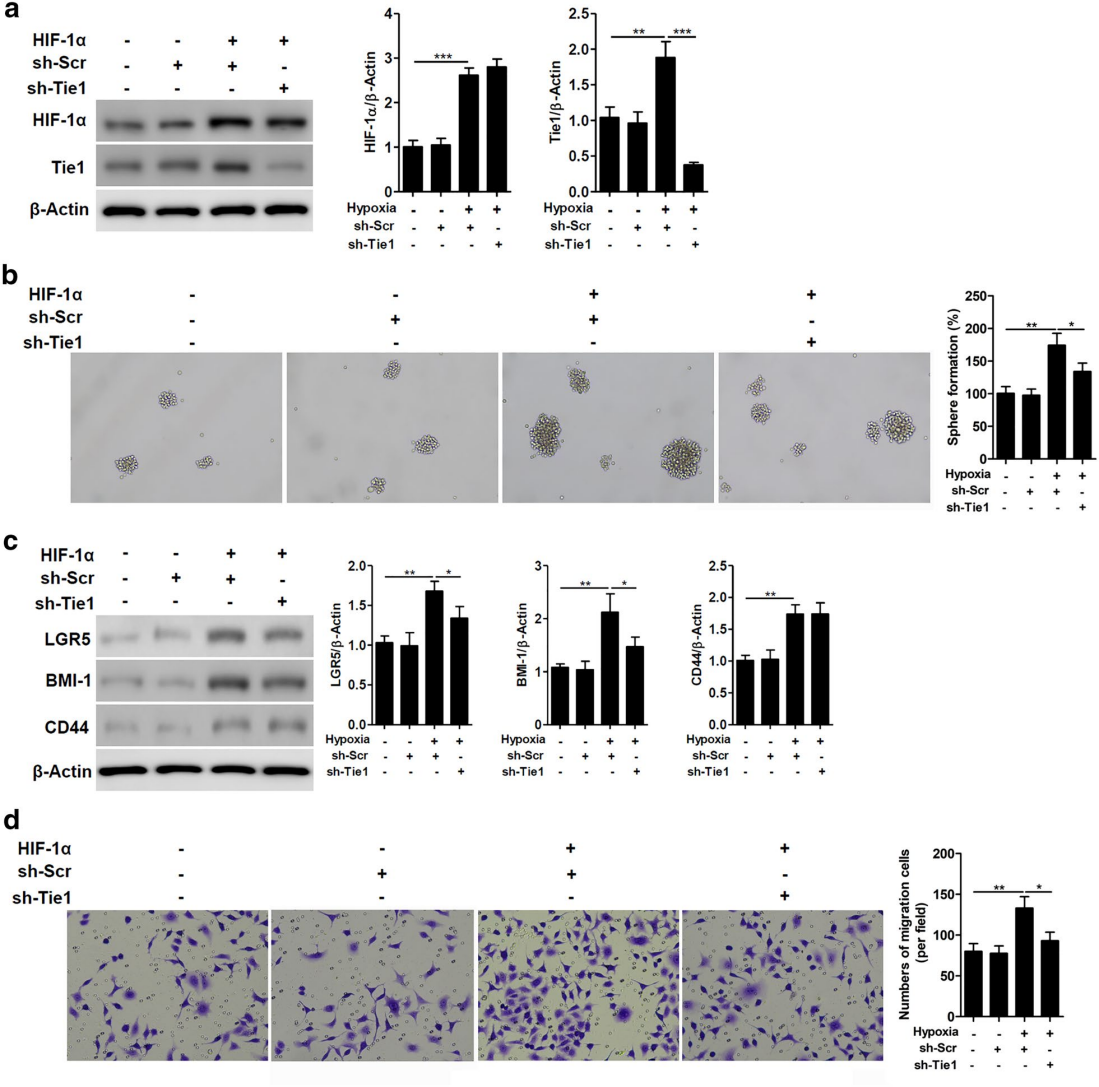
HIF-1α controls the stemness of NSCLC cells by regulating Tie1 expression. **a** Tie1 expression in A549 cells that express HIF-1α vector or HIF-1α and Tie1 shRNA vectors. Relative protein levels quantified by ImageJ and normalized to β-actin. **P < 0.01, ***P < 0.001. (n = 3). **b** Representative image of spheres in A549 cells that express a HIF-1α vector or HIF1α and Tie1 shRNA vectors. **c** The expression levels of stemness-related genes were determined by western blot analysis. Relative protein levels quantified by ImageJ and normalized to β-actin. *P < 0.05, **P < 0.01. (n = 3). **d** The migration ability of A549 cells was evaluated by Transwell assays. The number of migrated cells is shown in the bar graph. *P < 0.05, **P < 0.01. (n = 3)

Finally, we assessed whether HIF-1α could potentially control NSCLC cell stemness under hypoxia by regulating Tie1 expression. Tie1 expression is increased in A549 cells overexpressing HIF-1α (Fig. 6a). In addition, the formation of spheres seems to be predominately influenced by the expression of HIF-1α. Downregulating Tie1 reduces the number of spheres formed when HIF-1α is overexpressed but stemness remains significantly higher than the control (Fig. 6b). Similar results were found for the expression of cell division markers LGR5, BMI-1, and CD44 was stronger in cells overexpressing HIF-1α. However, the increase observed in the cells overexpressing HIF-1α decreased significantly in the absence of Tie1 (Fig. 6c). The migratory ability of A549 cells was also elevated in cells overexpressing HIF-1α in the presence of Tie1 (Fig. 6d). Although the number of migratory cells was reduced when Tie1 is downregulated, migration was still significantly higher than in control cells. From these results, we can conclude that HIF-1α controls the stemness of cells by regulating Tie1 expression in NSCLC.

## Discussion

Improving the outcome in NSCLC remains a challenge with growing evidence suggesting that hypoxia promotes the stemness of cells and resistance to therapy [24–27]. Moreover, a close relationship exists between the expression of HIF-1α and metastasis in NSCLC [28]. To clarify the mechanisms involved in hypoxia-related drug resistance and stemness and to identify potential therapeutic targets, we measured the impact of HIF-1α on the induction of Tie1 in NSCLC cells and the effects on drug resistance and stemness in vitro and in vivo. We found that when Tie1 was silenced there was a consequential increase in the sensitivity of NSCLC cells to cisplatin. High expression of Tie1 in NSCLC cells resistant to paclitaxel was discovered in an earlier large-scale microarray study [29]. The authors of this study identified several receptor tyrosine-protein kinases (RTK) in the drug resistance of NSCLC cells, including ERRB4, KIT, and Tie1, and suggested that the inhibition of RTKs may sensitize cancer cells to paclitaxel. Interestingly, they also found that NSCLC cell lines respond differently to different chemotherapeutic agents and the expression of Tie1 was relatively uninfluenced by docetaxel.

In the present study, we found that the expression of Tie1 was significantly elevated in response to hypoxia and that the down-regulation of Tie1 by shRNA could render cells more sensitive to cisplatin and reduce the stemness characteristics of cells. The involvement of Tie1 in the stemness of cancer cells has also been noted in another study [30]. La Porta et al. [30] studied Tie1 in tumor progression and found that the deletion of Tie1 in a mouse metastasis model prevented the extravasation of tumor cells into the lungs and reduced metastatic foci. In our study, hypoxia gave rise to increased drug resistance and an elevated level of genetic markers associated with stemness. Recent research supports the role of hypoxia in the stemness of cancer cells [31, 32]. In particular, HIF-1α has been found to promote the stemness of cancer cells through the regulation of several pathways, including PI3K/Akt/mTOR [26], Wnt/β‑catenin [33], and Notch [34] signaling. We used immunofluorescence to demonstrate that hypoxia influenced the expression of LGR5. Moreover, the levels of LGR5 are also influenced by PI3K/Akt/mTOR [35], Wnt/β‑catenin [36], and Notch signaling [37], suggesting that HIF-1α and hypoxia could also be involved in the regulation of LGR5. In a xenograft murine model of tumorigenesis using A549 NSCLC cells transfected with Tie1-shRNA, we found that the levels of Ki67 were reduced and that tumors were more sensitive to cisplatin. In a previous study, Gong et al. found that the suppression of Krüppel-like factors 5 (KLF5) could inhibit cisplatin resistance induced by the overexpression of HIF-1α by inactivating the PI3K/Akt/mTOR pathway in NSCLC cells [26]. Whether Tie1 is influenced by the PI3K/Akt/mTOR pathway has yet to be established.

In this study, we found that the induction of Tie1 in hypoxia is HIF-1α-dependent. By identifying two sites in the promoter of Tie1 with HRE binding consensus sequence (BDCGTV, B = C/T/G, D = A/G/T, V = G/C/A) [21], we were able to conduct luciferase assays and discovered that HIF-1α could upregulate Tie1 through interacting with both of these sites. ChIP analysis confirmed that HIF-1α could bind to the HRE sites in the promoter of Tie1. KLF5 is also known to co-immunoprecipitate with HIF-1α under hypoxic conditions in NSCLC [38]. HIF-1α has been recognized as a regulator of drug resistance in various drugs [39–41]. Under normoxic conditions, HIF-1α is located in the cytoplasm where it binds to von Hippel Lindau tumor suppressor (VHL) through the hydroxylation of proline residues and is subsequently degraded by the ubiquitin–proteasome system [42]. Under hypoxia, HIF-1α does not bind to VHL and is not degraded but it translocates to the nucleus where it forms a stable HIF-1 heterodimer with HIF-1β and acts as a transcription factor with genes controlled by HREs. Our results show an accumulation of undegraded HIF-1α in the cytoplasm of NSCLC cells under hypoxia. Therefore, in the hypoxic tumor microenvironment, genes that are involved in mechanisms related to stemness and drug resistance, such as Tie1 and KLF5, are artificially upregulated.

## Conclusion

Our results further support evidence that a hypoxic tumor microenvironment can increase the development of drug resistance and promote stemness characteristics in NSCLC. Interfering with the expression of Tie1 was able to increase sensitivity to cisplatin and reduce the migration and sphere-forming properties in cells. This study highlights the importance of counteracting hypoxia and proposes that Tie1 could be a potential target for increasing drug sensitivity and reducing stemness in the management of NSCLC.

## Data Availability

The data used to support the findings of this study are available from the corresponding author upon request.

## Abbreviations

NSCLC: Non-small cell lung carcinoma
HREs: Hypoxia-response elements
TIE: Tyrosine kinase with immunoglobulin and epidermal growth factor homology domains
HIF: Hypoxia-inducible factors
